# Near-Infrared Light-Activated Mesoporous Polydopamine for Temporomandibular Joint Osteoarthritis Combined Photothermal-Chemo Therapy

**DOI:** 10.3390/ijms24109055

**Published:** 2023-05-21

**Authors:** Qianli Li, Yi Hou, Pinyin Cao, Ruiye Bi, Songsong Zhu

**Affiliations:** State Key Laboratory of Oral Diseases, National Clinical Research Center for Oral Diseases, Department of Orthognathic and TMJ Surgery, West China Hospital of Stomatology, Sichuan University, Chengdu 610041, China

**Keywords:** mesoporous polydopamine, drug release system, NIR laser-responsive, controlled release, temporomandibular joint osteoarthritis

## Abstract

The treatments generally employed for temporomandibular joint osteoarthritis (TMJOA) involve physical therapy and chemotherapy, etc., whose therapeutic efficacies are impaired by the side effects and suboptimal stimulus responsiveness. Although the intra-articular drug delivery system (DDS) has shown effectiveness in addressing osteoarthritis, there is currently little reported research regarding the use of stimuli-responsive DDS in managing TMJOA. Herein, we prepared a novel near-infrared (NIR) light-sensitive DDS (DS-TD/MPDA) by using mesoporous polydopamine nanospheres (MPDA) as NIR responders and drug carriers; diclofenac sodium (DS) as the anti-inflammatory medication; and 1-tetradecanol (TD) with a phase-inversion temperature of 39 °C as the drug administrator. Upon exposure to 808 nm NIR laser, DS-TD/MPDA could raise the temperature up to the melting point of TD through photothermal conversion, and intelligently trigger DS release. The resultant nanospheres exhibited an excellent photothermal effect and effectively controlled the release of DS through laser irradiation to accommodate the multifunctional therapeutic effect. More importantly, the biological evaluation of DS-TD/MPDA for TMJOA treatment was also performed for the first time. The experiments’ results demonstrated that DS-TD/MPDA displayed a good biocompatibility in vitro and in vivo during metabolism. After injection into the TMJ of rats afflicted with TMJOA induced by unilateral anterior crossbite for 14 days, DS-TD/MPDA could alleviate the deterioration of TMJ cartilage, thus ameliorating osteoarthritis. Therefore, DS-TD/MPDA could be a promising candidate for photothermal-chemotherapy for TMJOA.

## 1. Introduction

Temporomandibular joint osteoarthritis (TMJOA) is a universal degenerative disease with high incidence, which impedes the normal function of temporomandibular joint (TMJ) and highly deteriorates patients’ quality of life [1,2]. Non-surgical therapies for TMJOA include physical therapy such as heat application [3], chemotherapy including oral medication with nonsteroidal anti-inflammatory drugs (NSAIDs) [4], and local injection of hyaluronic acid [5]. Although these forms of management, when used in combination, have been proven beneficial to relieve symptoms and prevent the progress of the osteoarthritis [6,7], their effects seem to be modest due to the side effects associated with the short-term effectiveness of physical therapy, poor targeting and gastrointestinal toxicity of oral medication [8], and the rapid clearing of injected agents. Therefore, alternative approaches need to be developed in order to improve the local inflammatory TMJ environment and enhance therapeutic efficacy.

In recent decades, photothermal technology (PTT) has attracted considerable attentions as a novel OA therapy through converting near-infrared light (NIR) to heat by tissue absorption [9,10]. To improve the efficient concentration of the photothermal effect at joints, exogenous photothermal agents with a distinct absorption spectrum are often applied to ensure that the photothermal effect has a marginal impact on the surrounding tissues and cells [11]. NIR-responsive agents consisted of gold nanospheres with anisotropy, biological transparency and a tunable NIR region [12,13,14,15], carbon-based nanomaterial [16,17], and organic nanomaterials such as NIR dyes (cyanine dyes, Prussian blue, etc.) [18,19]. Among the various NIR-responsive agents, polydopamine (PDA) has immense application as an excellent functional material for lower tissue absorption, efficient penetration of tissue, minimal scattering and ease of synthesis [20], as well as its outstanding biocompatibility, biodegradation, and adhesive properties [21]. Mesoporous PDA (MPDA), with great loading capacity of drugs and the photothermal ability to induce the light-heat conversion then trigger the drug release, has emerged as a promising drug delivery system (DDS) [22,23]. Studies have confirmed that MPDA-based DDS was efficacious in inhibiting the production of inflammatory cytokines such as prostaglandin E2 (PGE2) and cartilage degeneration, associated with osteoarthritis, almost all of which focus on the application to knees [24,25,26]. TMJs are intricate joints that connect the mandible and skull, facilitating essential functions such as mastication, deglutition, respiration, and speech despite their relatively small size [27]. Hence, it is not feasible to generalize the efficacy of drug-transported MPDA in treating knee OA to TMJOA.

Diclofenac sodium (DS), aimed at reducing the PGE2 level by the inhibition of cyclooxygenase [28], is one of the most effective oral NSAID interventions in diseases related to TMJ [29,30]. Because of its short half-life in plasma (1–2 h) and specific adverse events such as gastric ulcers and gastrointestinal bleeding [31], it is crucial to seek suitable conveyance to the designated location to diminish these side effects. Due to the poor water-solubility and negative charge of DS [32], the encapsulation and delivery technique need to be optimized in order to improve its loading in MPDA with negative charge. Therefore, we introduce 1-tetradecanol (TD) to the MPDA-DS delivery system, since TD interacts with the repulsive molecules and substances as a saturated aliphatic alcohol [33]. TD has been generally utilized as a gatekeeper in DDS, owing to its biocompatible thermosensitivity and phase-changing properties [34,35,36]. Thanks to TD, with a melting point of 38.8 °C (a little higher than the normal human body temperature), the whole system is sealed up under a physiological temperature so that the premature release of the drug is prevented. When the temperature is higher than 39 °C, drugs could be diffused in or out of the interior of MPDA through the fluid TD. A previous study [37] has indicated that a drug- and TD-loaded PDA system demonstrated a great potential for therapy of cancer. Furthermore, TD exhibited immunosuppressive effects on T cells [38] and inhibited inflammatory cell infiltration [39], which may improve the inflammatory microenvironment of TMJ. In this way, the application of TD being imported to drug-loaded MPDA (DS-TD/MPDA) achieve the dominated regulation of drug release via NIR irradiation. The potential for unpredicted and irresistible fluctuations in local drug concentrations could be reduced. An ingenious combination of photothermal and chemical therapies aiming to treat TMJOA “on-demand” has been demonstrated for the first time.

In this study, we design a dual photothermal-chemo drug-loaded nanoplatform aiming to innovatively treat TMJOA. The system utilizes MPDA as NIR photo-responsive carriers, and DS to manage inflammation with TD as gatekeepers. The controlled release of DS is achieved by subjecting the system to NIR irradiation. MPDA turn light energy to heat so that the surrounding temperature increasing above the melting point where TD changes from solid to liquid, facilitating the outflow of DS. The physicochemical properties of DS-TD/MPDA were characterized. Meanwhile, the photothermal characteristic and in vitro drug release of DS were detected. To examine the cytotoxicity of DS-TD/MPDA, they were incubated with ADTC chondrocytes cell lines in vitro. Finally, the therapeutic effect was examined by the injection of DS-TD/MPDA to arthritic TMJ of rat models. These results indicated the great potential of DS-TD/MPDA, which integrated multiple treatments simultaneously for the treatment of TMJOA by intra-articular injection.

## 2. Results and Discussion

### 2.1. Fabrication and Characterization of MPDA and DS-TD/MPDA

**Figure 1:**
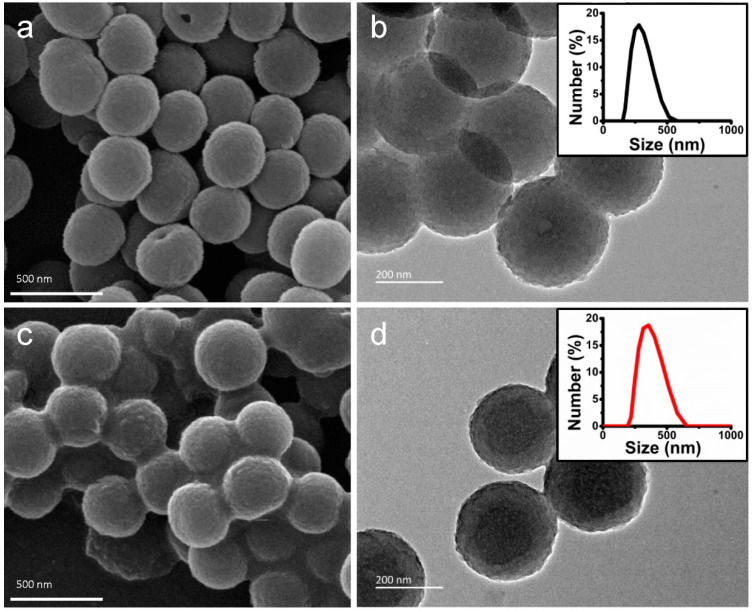
The morphology of the resulting nanospheres. (**a**) SEM image of MPDA. (**b**) TEM image of MPDA (inset: DLS of MPDA). (**c**) SEM image of DS-TD/MPDA. (**d**) TEM image of DS-TD/MPDA (inset: DLS of DS-TD/MPDA).

In this study, mesostructured polydopamine (MPDA) nanospheres were prepared as in a previous study [40] using F127 as a templet, TMB as a pore-enlarging agent, and dopamine as the source of nitrogen and carbon. The morphology of MPDA nanospheres and DS and TD-loaded MPDA were assessed by scanning electron microscopy (SEM) and transmission electron microscopy (TEM). Figure 1a showed that uniform MPDA nanospheres had an average particle size of 337 nm. The mesoporous structure of MPDA on the surface was clear in the observation of SEM. The TEM image also revealed that MPDA nanospheres possess a rough surface, similar to golf balls, with well-distributed pores exposed (Figure 1b). The mean particle diameter of DS-TD/MPDA was estimated to be 342 nm (Figure 1c,d), a bit larger than pure MPDA. Both of the SEM and TEM images displayed that the rough surface of nanospheres became blurred after coating with TD and DS. The average hydrodynamic diameter of MPDA and DS-TD/MPDA examined by dynamic light scattering (DLS) were similar to the measurements of TEM (inset of Figure 1b,d).

**Figure 2:**
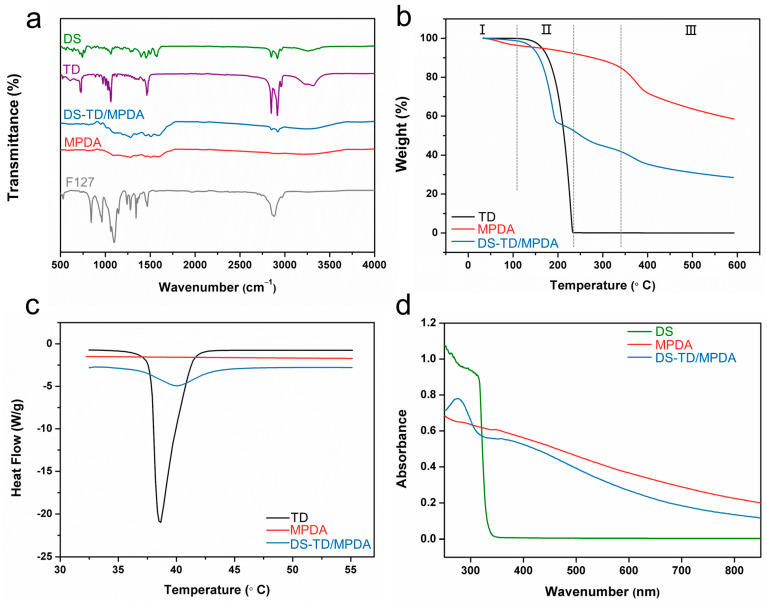
(**a**) FT-IR of F127, DS, TD, MPDA and DS-TD/MPDA. (**b**) TGA curves of TD, MPDA and DS-TD/MPDA. (**c**) DSC curves of TD, MPDA and DS-TD/MPDA. (**d**) UV-vis spectra of DS, MPDA and DS-TD/MPDA.

Fourier transform infrared (FTIR) spectroscopy analysis was conducted to determine if the template was removed and drugs were successfully loaded. As Figure 2a shows, there was no sign of the characteristic peak in F127 at 2881 cm^−1^ in the spectrum of MPDA, which demonstrated that F127 was successfully removed from MPDA after methanol reflux for 24 h extraction [41]. Compared to the FTIR spectrum of MPDA, it is obvious that DS-TD/MPDA possess characteristic peaks in DS and TD. The peaks emerging in the spectrum of DS-TD/MPDA at 1256 cm^−1^ and 1566 cm^−1^ could be the C-N stretching of aromatic amine and C=O stretching of carboxyl ion in DS [42]. The absorption at 1456 cm^−1^ is due to the stretching vibration of -CH2- groups of TD [43]. The peaks at 2846 cm^−1^ and 2912 cm^−1^, indicating the vibration and bending of -C-H groups of TD and DS, reappeared after reaction [44]. The peak in the range from 3200 cm^−1^ to 3330 cm^−1^ was attributed to the -OH vibration band. The spectrum of DS-TD/MPDA was similar to that of DS and TD. These results suggested that diclofenac sodium and phase change material were physically loaded into MPDA through hydrogen bonding rather than chemical interaction [45].

The results of the thermogravimetric analysis (TGA) were shown in Figure 2b, demonstrating a total weight loss of 42% in MPDA and 71.5% in DS-TD/MPDA. To understand the distribution of each component in the drug-load products, analysis was performed at various stages. Stage Ⅰ (28 °C–100 °C) witnessed a weight loss of less than 4% due to water evaporation for all samples. In Stage Ⅱ (100 °C–233 °C), pure TD experienced a weight loss of 99.6%, whereas MPDA and DS-TD/MPDA lost 4.7% and 47% of their total weight, respectively. The loading amount of TD was calculated to be 42.3%. Stage Ⅲ commenced from 335 °C, where MPDA exhibited a weight loss of 24% and DS-TD/MPDA had a loss of 11.5%. The mass difference at this stage implied that residual diclofenac molecules may have disrupted the carbonization process of polydopamine [41]. The melting temperatures of TD and synthesized nanospheres were evaluated using differential scanning calorimetry (DSC). As Figure 2c shows, no peak was observed in the curve of MPDA, while pure TD exhibited a sharp melting point at 38.6 °C. After the fabrication of DS-TD/MPDA, the emergence of the melting peak indicated the thermal phase-changing characteristic of the composite derived from TD. The melting point shifted rightwards to 40 °C and widened, suggesting that the liquid-solid conversion of TD was slightly restrained as a result of being contained within the pores of MPDA [46]. Based on the aforementioned results, the release behavior of DS-TD/MPDA, controlled by a phase-change material (TD), was initiated at 40 °C. The UV-vis absorption spectrum of MPDA exhibited a considerable capacity for absorption in the near-infrared range. The characteristic peak of DS distinguished at 276 nm appeared after the fabrication of DS-TD/MPDA (Figure 2d), which demonstrated the presence of NSAIDs.

### 2.2. Photo Characteristics of DS-TD/MPDA

**Figure 3:**
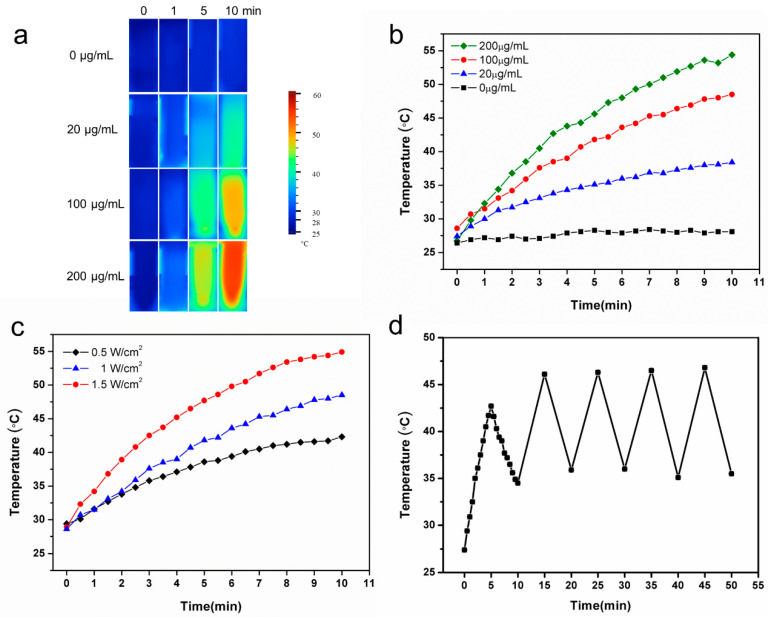
Photothermal effects of DS-TD/MPDA with laser irradiation (808 nm). (**a**) Thermal images of different concentrations DS-TD/MPDA in aqueous solution (1 W/cm^2^). (**b**) Temperature variation curve of different concentrations of DS-TD/MPDA (1 W/cm^2^). (**c**) Temperature variation curve of DS-TD/MPDA (100 µg/mL) of different power NIR laser irradiation. (**d**) Temperature variations of DS-TD/MPDA (100 µg/mL, 1 W/cm^2^) under NIR irradiation for five cycles.

As shown in Figure 3a,b, the photothermal images and the corresponding heating profiles of the DS-TD/MPDA after NIR irradiation showed that the photothermal effect was concentration-dependent. The changing temperatures of PBS were negligible, with increments of less than 2 °C during 10-min irradiation. Instead, the temperature of varying concentrations of DS-TD/MPDA solution gradually increased after illumination for 10 min, up to 38.4 °C, 48.5 °C, and 54.4 °C at the concentration of 20, 100, and 200 µg/mL, respectively. Moreover, the photothermal performance of DS-TD/MPDA was related to the laser density (Figure 3c). As the laser power increased, the temperature of DS-TD/MPDA suspension at 100 µg/mL concentration rose accordingly. The photothermal effect of DS-TD/MPDA suspension (100 µg/mL) was stable after five cycles of repeated NIR laser irradiation (Figure 3d). These results indicated that DS-TD/MPDA was a potential agent for TMJ hyperthermia based on its photothermal stability.

### 2.3. Loading Capacity and Release of DS

**Figure 4:**
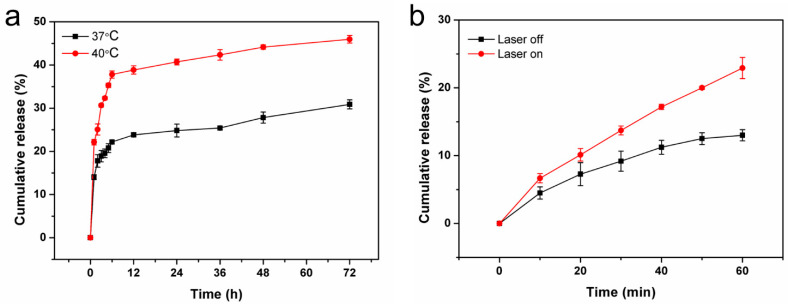
(**a**) DS release behaviors of DS-TD/MPDA at 37 °C and 40 °C. (**b**) DS release behaviors of DS-TD/MPDA under the 808 nm laser irradiation, 1 W/cm^2^.

The amount of loaded DS was calculated to be 2.58%. To investigate the controlled release profiles for DS out of DS-TD/MPDA, thermal stimulation and NIR irradiation were performed. As shown in Figure 4a, 23.7% and 38.8% of DS release at 37 °C and 40 °C, respectively, occurred for the first 12 h. The percentage of DS rose to 30.8% and 45.9% within 72 h. In addition, the release of DS rapidly increased for 1 h after being triggered by NIR laser (Figure 4b). These results demonstrated that DS-TD/MPDA was effective in ensuring drug release through a photothermal response.

### 2.4. Cytotoxicity Detection of DS-TD/MPDA

**Figure 5:**
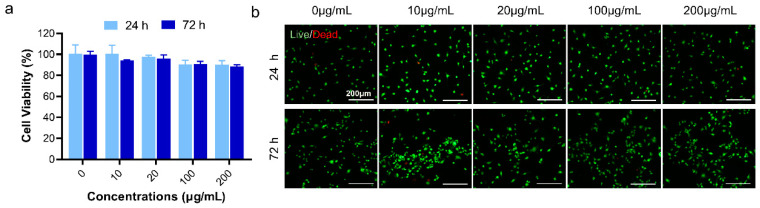
Cell viability assay of DS-TD/MPDA after 24 and 72 h incubated with ATDC5 cells. (**a**) CCK-8 assay. (**b**) Live/Dead staining images.

To examine the cytotoxicity of DS-TD/MPDA, Cell Counting Kit-8 (CCK-8) assay and Live/Dead assay were performed at 24 and 72 h after ADTC5 cells incubated with various concentrations of DS-TD/MPDA (0, 10, 20, 100, 200 µg/mL). In CCK-8 test (Figure 5a), the cell viability of ADTC5 cells incubated with DS-TD/MPDA at 10 µg/mL and 20 µg/mL concentration were approximately over 97% at 1 and 3 d. Even at higher concentrations of up to 200 µg/mL, no significant difference was found between control and drug-load MPDA in terms of cell viability and proliferation. Furthermore, living/dead cells were visualized using the live/dead staining with calcein acetoxymethyl (calcein-AM) and propidium iodide (PI). As revealed in Figure 5b, most ADTC5 cells stay alive (green cells) when cocultured with different concentrations of DS-TD/MPDA at 24 and 72 h. A few dead cells (red cells) were captured at all concentrations, including 0 µg/mL after 24 h cell culturing. At 72 h, the cell density of every group was obviously raised compared to that at 24 h. The number of dead cells remained limited. These results demonstrated that DS-TD/MPDA was a biocompatible photothermal-irritated drug carrier for the chondrocytes.

### 2.5. Therapeutic Effectiveness of DS-TD/MPDA for TMJOA

**Figure 6:**
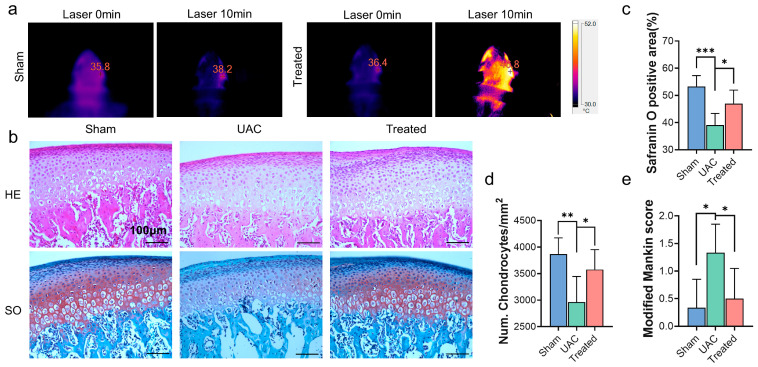
(**a**) In vivo photothermal performance of TMJ with and without injection of DS-TD/MPDA (100 μg/mL) under NIR laser irradiation (808 nm) before and after 10 min. (**b**) H&E staining and Safranin O-fast green staining of the condylar cartilage of rats after 2 weeks’ treatment with different samples. (**c**) The Safranin O positive area of the articular cartilage. (**d**) The number of chondrocytes in articular cartilage. (**e**) The modified Mankin score of the articular cartilage. * *p* < 0.05; ** *p* < 0.01, *** *p* < 0.001.

Temporomandibular joints (TMJs) are complicated and significant organs connecting mandible and skull, playing a critical role in orofacial functions such as mastication, deglutition, respiration and speech [47]. Dysfunction of TMJs could cause common clinical problems, for example, temporomandibular joint osteoarthritis (TMJOA) with pain, joint clicking, limited mouth opening and chewing difficulties. Efforts to improve the therapeutic effect of TMJOA treatment have been ongoing for decades. Previous study [48] showed biodegradable poly(DL-lactic-co-glycolic acid) (PLGA) microparticles (MPs) encapsulating anti-inflammatory small interfering RNA reduced inflamed changes in TMJs. Lipid carrier formulation for the intra-articular administration of naproxen was proved to significantly reduce the migration of leukocytes in inflamed TMJs [49]. However, the DDS mentioned above were insufficiently advanced to release drugs according to the needs of patients experiencing pain and oral maxillofacial dysfunctions. Infrared light-emitting therapy was easily accessible and manipulated at home, which effectively alleviating pain and restoring mandibular function in TMJ disease [50]. Thus, we synthesized a NIR-manipulated drug delivery system and release drugs on demand. The temperature of TMJ injected with DS-TD/MPDA was shown in Figure 6a, which rose from 36.4 °C to 46.8 °C after 808 nm NIR irritation for 10 min. The change of temperature of TMJ without injection was less than 2 °C under laser exposure.

A unilateral crossbite (UAC) rat model was proved to effectively induce TMJ osteoarthritis in a previous study [51], which imitated one of the main causes of OA by loading abnormal biomechanical force on TMJs. Metal tubes were installed on the mandibular anterior tooth of Sprague-Dawley rats to alter the occlusion. Thus, TMJOA was developed with the intact structures of TMJ, facilitating the injection and residence of materials in articular space. The histology of cartilage tissues was evaluated by hematoxylin-eosin (H&E) staining and Safranin O-fast green staining 2 weeks after UAC generation (Figure 6b). We observed that the number of chondrocytes in condylar cartilage and the Safranin O positive area were significantly reduced in UAC rats compared with the other two groups (Figure 6c,d). The group treated with DS-TD/MPDA injection and laser exposure exhibited less cartilage degeneration, although there was little difference in comparison to the sham group. The modified Mankin score was chosen to evaluate the destruction of cartilage (Figure 6e). The modified Mankin score of the UAC group was obviously higher than the sham group and treated group (average of 1.2 vs. 0.3–0.4). In general, these findings indicated that the NIR-responsive DS-TD/MPDA drug-loaded platform alleviated cartilage degradation in temporomandibular joint osteoarthritis.

### 2.6. Biocompatibility of DS-TD/MPDA in Vivo

**Figure 7:**
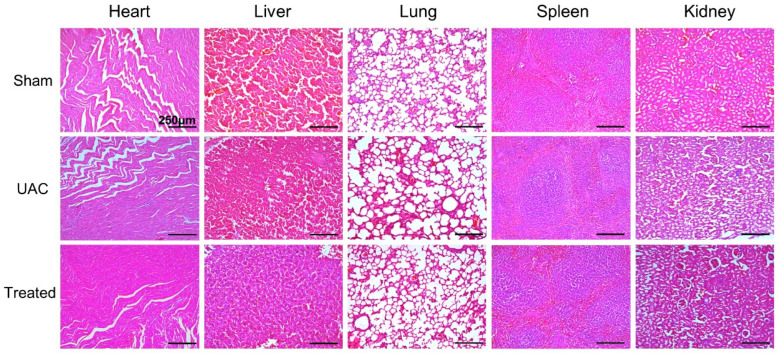
Histological images of major organs (heart, liver, lung, spleen and kidney) of rats with H&E staining.

HE staining images of major organs of rats, including heart, liver, lung, spleen, and kidney, were shown in Figure 7. No obvious pathological feature was found in these organs when DS-TD/MPDA was injected into the joint cavity. It was evaluated that DS-TD/MPDA had few toxic side effects during metabolism on the body.

## 3. Materials and Methods

### 3.1. Materials

Dopamine hydrochloride, Pluronic F127, and 1, 3, 5-Trimethylbenzene (TMB), ammonia solution, Diclofenac Sodium (DS), and 1-tetradecanol (TD) were purchased from Aladdin Industrial Corporation (Shanghai, China). Methanol was purchased from Kelong Chemical Corporation (Chengdu, China). Fetal bovine serum (FBS), penicillin–streptomycin (PS) and Dulbecco’s modified eagle medium (DMEM) were purchased from Gibco Corporation (Grand Island, NY, USA). Cell counting kit-8 (CCK-8) from APExBIO (Houston, TX, USA) and live/dead staining tested by a live/dead cell kit (KeyGEN BioTECH, Nanjing, China) were used as the protocols recommended. All other chemical reagents were analytical grade and used without further purification unless indicated. Rats were purchased from Dashuo animal company (Chengdu, China). Animals were housed in the experimental animal center of the university with sufficient water and food at 25 °C with 40% humidity under a 12 h light/dark cycle.

### 3.2. Synthesis of MPDA

MPDA were synthesized and modified based on a previous study [40]. In brief, 1 g of F127, 1 g of dopamine hydrochloride and 1 mL of TMB were dispersed in a solution of 50 mL ethanol and 50 mL water. After 5 min of ultrasonication, 0.8 mL of concentrated ammonia solution (28 wt%) was added dropwise into the milk-like mixture under continuous stirring for 8h. Black product was collected by centrifugation (12,000 rpm), then washed with ethanol and water three times. To remove the F127 templates, product was re-dispersed in 200 mL methanol for 24 h extraction.

### 3.3. Synthesis of DS and TD Loaded MPDA (DS-TD/MPDA)

The process of loading DS and TD onto MPDA was conducted in accordance with a previous study [52] with slight modifications. For the preparation of DS-TD/MPDA, the freeze-dried MPDA nanospheres (20 mg) were dispersed in 3 mL methanol by ultrasonication. At the same time, TD (15 mg) and DS (10 mg) was mixed and stirred in 2 mL methanol at 60 °C. Then, MPDA nanospheres were added to the mixture after sonication for 5 min. The gradual diffusion of liquid TD molecules combined with DS resulted in their steady flow into the MPDA hollow pores via the evaporation of methanol. After stirring for another 6 h, 5 mL of hot water (80 °C) was added, precipitates were centrifuged (12,000 rpm) and washed by cold water to remove excess TD and DS, and the product was freeze-dried for further use.

### 3.4. Characterization

The morphology of these products was investigated using scanning electron microscopy (SEM, INSPECT F, Thermo Fisher Scientific, Waltham, MA, USA) and transmission electron microscopy (TEM, JSM7500F, Hitachi, Tokyo, Japan). Dynamic light scattering (DLS) was analyzed using SZ-100 Nanoparticle Size Analyzer (Horiba, Kyoto, Japan). Fourier transformation infrared spectra (FT-IR) were measured at room temperature on a spectrometer (INVENIO R, Bruker, Karlsruhe, Germany). Thermogravimetric analysis (TGA) and differential scanning calorimetry (DSC) using a thermal analyzer (TGA/DSC2, METTLER TOLEDO, Zurich, Switzerland) were conducted by heating the samples from 32 to 600 °C at a heating rate of 10 °C/min under Ar atmosphere with a flow rate of 100 mL/min. UV-vis spectra was recorded from 250–850 nm using a UV–vis spectroscopy (UV-4800, Unicosh, Shanghai, China).

### 3.5. Photothermal Properties of the DS-TD/MPDA

As-prepared DS-TD/MPDA was suspended in PBS solution with gradient concentrations (0, 20, 100, and 200 μg/mL) and exposed on an 808 nm laser (1 W/cm^2^) for 10 min. Then, 100 μg/mL of DS-TD/MPDA aqueous suspension was irradiated by 808 nm laser at 0.5, 1, 1.5 W/cm^2^ lasting for 10 min. Laser ON/OFF cycles were repeated 5 times to evaluate the photostability of the drug-loaded nanospheres. The temperature changes of DS-TD/MPDA suspension were recorded by IR thermal imaging (Ti401 PRO, Fluke, Everett, WA, USA) in real time.

### 3.6. Measurement of Drug Loading and Release

DS-TD/MPDA was resuspended in acetone to extract DS by gently heated and stirred for 2 h. After centrifugation, the surfactant was diluted with the PBS solution and analyzed for DS by UV spectrophotometer at 276 nm. The loading capacity (%) of DS was estimated as following equation: loading capacity (%) = (the amount of DS loaded/the amount of DS-TD/MPDA) × 100. The loading amount of TD was evaluated by TGA.

To investigate the release of DS stimulated by temperature and laser exposure, DS-TD/MPDA suspended in PBS solution (100 μg/mL) in dialysis bag (12,000–14,000 Da MWCO, Yuanye, Shanghai, China) was directly heated in 37, and 40 °C with 100 rpm shaking for a 72-h period. The 1 mL sample was taken from the release medium at each specified time, then replaced with the same amount of fresh PBS. DS-TD/MPDA also went through five laser on/off circles: five min laser-on followed by five min laser-off. DS concentrations were determined by UV spectrophotometer at 276 nm.

### 3.7. In Vitro Cytotoxicity Assay

The cytotoxicity of DS-TD/MPDA against ATDC5 cells was evaluated using CCK-8 and live/dead cell viability assay. Specifically, ATDC5 cells were cultured in DMEM with 10% fetal bovine serum (Gibco, Grand Island, NY, USA), and 1% penicillin/streptomycin (Gibco) in a 12-well plate (Corning, Corning, NY, USA), at a density of 2 × 10^5^ cells per well. The DS-TD/MPDA nanospheres were suspended in culture medium at different concentrations (0, 10, 20, 100, and 200 μg/mL). ATDC5 cells were co-incubated with the culture medium contained DS-TD/MPDA nanospheres for 24 and 72 h, respectively. The cells were carefully rinsed by PBS two times before incubation with 10% CCK-8 solution for 1 h, followed by the determination of absorbance at 450 nm using a microplate reader (Varioskan LUX, Thermo Fisher, Waltham, MA, USA). For live/dead cell viability assay, ATDC5 cells were stained with calcein acetomethoxyl (calcein-AM) and propidium iodide (PI) for 30 min, followed by PBS rinse 2 times. Images of cells were captured by fluorescence microscope (Leica DMi8, Leica, Wetzlar, Germany), the λex/λem of calcein-AM and PI are 499/515 nm and 495/635 nm, respectively.

### 3.8. Establishment and Treatment of the Unilateral Anterior Crossbite (UAC) Rat Model

The UAC stimulation was applied as Zhang [51] described previously. Fifteen 8-week-old male Sprague-Dawley (SD) rats were divided into 3 groups randomly: sham group (Sham); UAC group; and UAC + laser group (Treated group). Syringe needles (diameter = 4 mm; length = 2–4 mm) were used to made metal tubes with 135° labial inclination. For UAC rats, metal tubes were bonded to right pair of mandibular incisors with the zinc phosphate cement after rats were anesthetized. The rats of sham group were only anesthetized, without putting tubes on the incisors. The treated rats were forced to crossbite as well, following injection with 20 μL DS-TD/MPDA (100 μg/mL) into the right TMJ capsule. After injection for 24 h, the right TMJ joint was exposed to local infrared laser radiation (808 nm, 1.5 W/cm^2^) for 8 min, twice a week. Two weeks after model generation, SD rats were sacrificed and fixed in 4% PFA (4% paraformaldehyde in 0.01 M PBS, pH 7.4) for 24 h. Right-side TMJ blocks were trimmed and decalcified in 15% EDTA solution (15% ethylenediaminetetraacetic acid disodium salt solution, pH 7.5) for 28 days at room temperature. Then, the TMJ blocks were paraffin embedded and serial sections were cut in the sagittal plane with the thickness of 6 μm. The main internal organs of the rats (heart, liver, lung, spleen, and kidney) were collected, sliced and stained with HE using the same method.

### 3.9. Histopathological Analysis

To estimate the degeneration of condylar cartilage, hematoxylin and eosin staining (HE, Biosharp, Hefei, China), and Safranin O/fast green (SO) staining (SO&FG, Solarbio, Beijing, China) were performed. Images were captured by light microscope (Leica DM2000, Leica, Wetzlar, Germany). The number of chondrocytes was counted in HE staining. SO positive area was calculated by the Image 6.0 image analysis system. A modified Mankin score system was used to assess the severity of cartilage arthritis [53]. According to the number and morphology of chondrocytes, the safranin O positive region of cartilage matrix, and cartilage integrity, normal cartilage scored 0–1 point. A total of 2–4 points were assigned to mild osteoarthritis.

### 3.10. Statistical Analysis

All statistical analyses were performed using Prism 9.0 software (GraphPad, San Diego, CA, USA). One-way analysis of variance (ANOVA) was used to compare the differences between the three groups (* indicate *p* < 0.05; ** *p* < 0.01, *** *p* < 0.001).

## 4. Conclusions

In this study, we successfully synthesized a novel NIR-sensitive drug-loaded system aimed at TMJOA therapy. Experimental results indicated that DS-TD/MPDA was compounded through a process of physical interaction. The modulation of drug release was smartly controlled utilizing the thermal properties of phase change material (TD) during the light-heat conversion of MPDA upon exposure to a laser. Moreover, the nanoplatform could effectively relieve the inflammation of TMJ and inhibit condylar cartilage destruction by the intra-articular injection of DS-TD/MPDA with laser irradiation. These findings suggest that such a photothermal- and chemotherapy-associative nanoplatform could be a promising strategy for TMJOA.

## Data Availability

Not applicable.
